# Urban Land Expansion from Scratch to Urban Agglomeration in the Federal District of Brazil in the Past 60 Years

**DOI:** 10.3390/ijerph19031032

**Published:** 2022-01-18

**Authors:** Zhichao Li, Helen Gurgel, Minmin Li, Nadine Dessay, Peng Gong

**Affiliations:** 1 Key Laboratory of Land Surface Pattern and Simulation, Institute of Geographic Sciences and Natural Resources Research, Chinese Academy of Sciences, Beijing 100101, China; 2 Department of Geography, University of Brasilia, Brasilia 70910-900, Brazil; helengurgel@unb.br; 3 Key Laboratory of Urban Land Resources Monitoring and Simulation, Ministry of Natural Resources, Shenzhen 518060, China; limm@szu.edu.cn; 4 Technology Innovation Center of Territory & Spatial Big Data, MNR & Research Institute for Smart Cities, School of Architecture and Urban Planning, Shenzhen University, Shenzhen 518060, China; 5 ESPACE-DEV, UMR 228 IRD/UM/UR/UG, Institut de Recherche pour le Développement, 34093 Montpellier, France; nadine.dessay@ird.fr; 6 Ministry of Education Key Laboratory for Earth System Modelling, Department of Earth System Science, Tsinghua University, Beijing 100084, China

**Keywords:** purpose-built city, urban expansion, spatial pattern, Brazil

## Abstract

Empirical studies of urban expansion have increased rapidly in recent decades worldwide. Previous studies mainly focused on cities in China, the United States or African countries, with Brazilian cities receiving less attention. Moreover, such studies are rare in purpose-built cities. Taking the urban expansion from scratch (1960) to urban agglomeration (2015) in the Federal District of Brazil (FDB) as an example, this study aims to quantify the magnitude, patterns, modes, types and efficiency of urban land expansion and attempts to reveal some implications within sustainable urban expansion thinking. Annual expansion, landscape metrics, local Moran’s I index, area weighted mean expansion index, and land-use efficiency were computed. The suitability of diffusion–coalescence theory and the impact of population growth and urban development policies on urban expansion were discussed. Urban land continuously expanded and became more fragmented during 1960–2015, which mainly occurred in SSW and WSW directions. Urban land evolved in a polycentric way. Edge expansion was identified as the stable contributor, and the importance of infilling and spontaneous growth alternated. Urban expansion in this region supported the diffusion–coalescence theory. Population growth promoted urban expansion, and the creation of peripheral urban nuclei and their development were associated with the urban expansion and the changes in urban land structure. This study adds new empirical evidence of urban expansion to Brazil urbanization, and compact urbanization, population control, and efficient urban land use should be considered in the future.

## 1. Introduction

With global urbanization, more than half of the world’s population were living in cities in 2018, and this proportion is expected to reach 68% by 2050 [1]. Urban expansion and changes in urban land structure have social, economic, ecological, and environmental impacts on urban populations and on urban sustainable development [2,3,4,5]. The Sustainable Development Goal (SDG) target 11.3 underlines the goal of enhancing sustainable urbanization in all countries by 2030. Implementing empirical studies of urban expansion permits to provide the baseline information of urban land-use change, enabling to reveal some implications for urban sustainable development [6,7].

A better understanding of urban expansion of planned cities could make a timely contribution to the literature, especially in the context of numerous new planned cities worldwide. Previous studies mainly focused on cities in China, the United States or African countries [7,8,9,10,11]. In contrast, although intense urban expansion has occurred in Brazil in recent decades, with close to 87% of the total population living in cities in 2018, Brazilian cities receive less attention. The Federal District of Brazil (FDB), one of the federative districts in Brazil, was created to house the capital, Brasilia, which is a purpose-built city founded in 1960. Since then, significant urban expansion occurred in the FDB, and it has become the third urban agglomeration in Brazil. However, urban expansion in the FDB is not well studied yet, and only a few studies involved the FDB while comparing the urban expansion of many urban areas worldwide [12,13,14]. An understanding of urban expansion from scratch to urban agglomeration in the FDB is needed to provide new evidence of Brazilian urbanization, which should not only be represented by the megacities, such as Rio de Janeiro and São Paulo.

Recent empirical studies of urban land expansion often involved the following aspects: urban expansion magnitude [6,11,15], urban development modes (e.g., monocentric or polycentric) [6,16], urban land patterns [8,11,15,17], urban expansion types [6,9,12], urban expansion theory [15,17], and land-use efficiency [18].

In analyses of urban land patterns, previous studies used various landscape metrics and various spatial analysis scales, such as the entire study area, the concentric rings, the different geographic sectors, or the areal units (e.g., sectors or segments) formed by the intersect of concentric rings and geographic directions [8,15,19,20,21,22,23,24,25,26,27,28]. However, despite the extensive use of landscape metrics in previous studies, an evaluation of their suitability to urban expansion data and specific spatial analysis units has been less considered. In fact, except for the objectives of the study and the interpretability of the landscape metrics (i.e., whether or not the interpretation is easy) [21,29], the behavior of the metrics also should be considered, as most metrics vary with scale-dependent characteristics (e.g., the spatial scales used to compute them) and with the intrinsic features of the landscape spatial pattern (e.g., the percentage of a landscape type) [30].

In terms of urban expansion types, many studies computed the land expansion index (LEI) to reflect the spatial relationships between newly expanded patches and pre-existing patches [8,15]. The new urban patches can be categorized into three types, namely, infilling, edge-expansion and spontaneous growth, and their relative dominance can provide an insight into urban land form (e.g., compact or dispersal) [31]. However, there are debates on how the dominance of urban expansion types change over time and space [6]. The FDB, with urban expansion from scratch to urban agglomeration in a few decades, might provide new evidence for such debates.

In analyses of urban expansion towards sustainable urban expansion, several indices have been developed to quantify how efficiently cities use land in terms of population growth, economic performance, and environmental benefits [6]. In fact, the SDG indicator 11.3.1 is defined as the ratio of the land consumption rate to population growth rate, used to understand the relationship between urban land and population [18,32]. Examining the efficiency of urban expansion from scratch to urban agglomeration could provide new evidence for local sustainable urban development.

Regarding the urban expansion theory, a common method is interpreting the temporal changes in landscape metrics and/or the dominance of urban expansion types [8,15,17,33,34]. In this theoretical model, the urban expansion process can be defined as an oscillation between the diffusion phase and coalescence phase [33,34]. The diffusion phase refers to a dispersed development of newly expanded patches, and the coalescence phase refers to infilling in the urban complex or the expansion outward from the existing urban patches. However, the suitability of this two-step cycle is under debate, as it highly depends on the form (i.e., polycentric or monocentric) and history of the city [35]. Due to the particular origin and rapid urban expansion of the FDB, testing the urban expansion theory in this region might add additional evidence for this debate.

In this context, focusing on urban land expansion in the FDB in the past 60 years, this study aims to answer three questions: (1) What evidence can be provided by the urban expansion in the FDB? (2) Is the diffusion–coalescence urban expansion theory suitable for urban expansion in the FDB? (3) How did population growth and urban development policies affect urban expansion in the FDB?

This study quantified the magnitude, patterns, modes, types, and efficiency of urban land expansion based on a comprehensive set of indicators to answer the first question. Based on quantitative results, this study discussed the temporal dynamics of the pattern and types of urban expansion, and the impact of urban expansion efficiency and the timing and place of urban development policies on urban expansion to answer the second and third research questions, respectively.

## 2. Materials and Methods

### 2.1. Study Area and Data Sources

**Figure 1:**
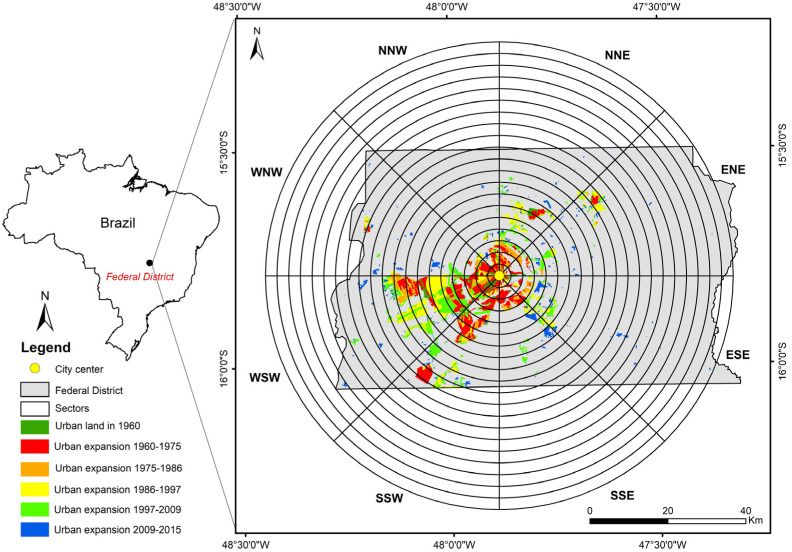
The administrative border (grey polygon) of the FDB, multi-date urban expansion data, and the concentric buffers–geographic directions system.

This study was carried out in the FDB, which is located on the central-western highlands in Brazil, with an area of approximately 5760 km^2^ (Figure 1). It is reserved for Brasilia, the new capital of Brazil founded in 1960. Brasilia is a planned city and its initial layout was proposed by Lucio Costa. Its central area was named as a World Heritage Site in 1987 by the United Nations Educational, Scientific, and Cultural Organization (UNESCO) due to its artistic urban planning and modernist architecture [36]. The city was expected to transform into a sustainable metropolis. However, in the past decades, significant urban structural changes, population growth, and various urban development policies occurred in the FDB [37]. According to the Brazil Institute of Geography and Statistics (IBGE), the population continuously increased from 139,796 people in 1960 to 3,015,268 people in 2019. Today, the FDB is the third largest metropolis in Brazil, which is divided into 33 administrative regions and is characterized by spatio-economic inequalities, such as lack of infrastructure in new urban land, low-income people concentrated in satellite towns, and fragmented landscapes and transportation. Thus, the FDB could be an ideal study area to provide a comprehensive understanding of rapid urban land expansion from a city’s origin to urban agglomeration, and examine urban expansion toward sustainable development.

The multi-date urban expansion data downloaded from the FDB Geoportal website (https://www.geoportal.seduh.df.gov.br, accessed on 1 December 2019) were used as input data for characterizing the urban expansion of the FDB. The data were produced by the State Secretariat for Housing and Urban Development of the Federal District, Brazil (SEDUH-DF) by manually vectorizing the mosaics of the high-resolution aerial photographs taken by Brasilia Real Estate Company, Federal District, Brazil (Terracap) and urban plans drawn up by SEDUH. Urban land in this dataset refers to the developed areas, including residential, industrial, commercial, transportation, administration/public services, and green space. In this study, due to the data availability, we focused on the urban land in 1960 and the expanded land for 1975, 1986, 1997, 2009, and 2015 (Figure 1). According to World Population Review, the population of Brasilia for 1960, 1975, 1986, 1997, 2009, and 2015 was 136,643, 827,361, 1,615,853, 2,549,093, 3,624,703, and 4,168,288, respectively.

### 2.2. Two Types of Spatial Scales

To quantify urban expansion in the FDB, we used two types of analysis: an analysis within the FDB border and an analysis based on the concentric buffers–geographic directions system (Figure 1). The former approach permits to provide the general information of urban expansion within the entire study area, and the latter approach integrates the concentric rings and geographic directions, and permits to provide a better comprehension of the characteristics of the urban expansion in different locations of cities that are often different [29,38,39,40]. Here, we first created twenty concentric rings at intervals of 3 km around the center of Brasilia (i.e., the center point defined at the beginning of the establishment of Brasilia) until the largest ring covered all urban land patches in 2015. Each ring was then divided into sectors located in eight geographic directions (i.e., NNE, ENE, ESE, SSE, SSW, WSW, WNW, and NNW). Finally, in each direction, twenty sectors of different sizes were generated. As shown in Table 1, urban expansion magnitude and urban land patterns were both evaluated at two types of spatial scales. The types, modes, and efficiency of urban expansion were analyzed within the entire study area. The detailed steps are presented hereafter.

### 2.3. Urban Expansion Magnitude

To evaluate the urban expansion magnitude, we computed the annual expansion (AE) of urbanized land within the entire study area and sectors (Figure 1) for five time periods (1960–1975, 1975–1986, 1986–1997, 1997–2009, and 2009–2015) as follows [8,17]:

(1) $$\mathrm{AE}=\frac{U_{t2}-U_{t1}}{T},$$

where *T*, *U_t_*_1_ and *U_t_*_2_ represent the time period, the urban land area at the start of the time period, and the urban land area at the end of the time period, respectively.

### 2.4. Urban Land Patterns

The description of a landscape-by-landscape metric computation includes two aspects: (1) compositional patterns, referring to the abundance and variety of patch types in a landscape; and (2) configurational patterns, a broader and more vague notion related to the spatial character, arrangement, and context of the patches in the landscape [41]. In this study, considering our objective and the interpretability of the metric, we selected six commonly used and easily interpreted landscape metrics (Table 2) from the literature based on the following two reasons: (1) they characterize the main urban land patterns, including the area, edge, shape, isolation, and subdivision of urban patches; and (2) they were commonly used in the test of diffusion–coalescence urban expansion theory, and their temporal variations (e.g., increasing and decreasing) in the model of diffusion–coalescence urban expansion theory are known [34]. Moreover, class-level computation of landscape metrics permits to measure the spatial patterns of a focal landscape type [42]. Therefore, in this study, the landscape metrics were calculated at the class level with an 8-connectivity implementation of the algorithm within the whole study area and each sector (Figure 1), using Fragstats software 4.2 (Amherst, MA, USA).

In the analysis based on the concentric buffers–geographic directions system (Figure 1), we first used the β-score and γ-score to examine the ability of the metrics to capture the changes in urban expansion over time (i.e., 1960, 1975, 1986, 1997, 2009, and 2015) and space (i.e., the sectors of different sizes), respectively. For each metric, we computed a β-score per year and a γ-score per sector. This method was adopted from the multiscale analysis of the behavior of the landscape metrics [43], the scalogram method used for studying the effects of the different spatial extents of the area of urban land using a series of concentric circles with increasing radii [44], and the rank-based landscape metric selection process used for spatial land cover pattern monitoring [45,46]. Then, all the metrics were ranked in descending order according to the mean value of β-scores and the mean value of γ-scores, respectively. Effective metrics should have higher values of the two scores, reflecting the better suitability to recognize the urban expansion patterns using our multi-date urban expansion data and spatial scales.

(2) $$\beta =\frac{\left(Max\left(S_{1},\ldots ,S_{m}\right)-Min\left(S_{1},\ldots ,S_{m}\right)\right)}{Max\left(S_{1},\ldots ,S_{m}\right)},$$

where $Max\left(S_{1},\ldots ,S_{m}\right)$ and $Min\left(S_{1},\ldots ,S_{m}\right)$ are the maximum and minimum values of a metric in each sector, and m represents the number of sectors. A total of 160 sectors (i.e., *m* = 160) were included in the β-score computation.

(3) $$\gamma =\frac{\left(Max\left(Y_{1},\ldots ,Y_{n}\right)-Min\left(Y_{1},\ldots ,Y_{n}\right)\right)}{Max\left(Y_{1},\ldots ,Y_{n}\right)},$$

where $Max\left(Y_{1},\ldots ,Y_{n}\right)$ and $Min\left(Y_{1},\ldots ,Y_{n}\right)$ are the maximum and minimum values of a metric in each year, and n represents the number of years. A total of 6 years (i.e., *n* = 6) were included in the γ-score computation.

### 2.5. Urban Expansion Modes

The local Moran’s I index [47] has been used in a case of a polycentric urban expansion to reveal the temporal changes of urban land agglomeration [6]. In this case, a spatial analysis grid was defined, and the area of urban land per grid cell was computed. Local Moran’s I index was then computed based on the grid, and only the grid cells with a *p*-value ≤ 0.05 were presented for displaying the agglomeration of urban land. The spatial agglomeration of urban land was indicated by high–high clusters that would be the grid cells with a high area of urban land surrounding by other grid cells with a high area of urban land. Low–high indicates the grid cells surrounded by other grid cells with a higher area of urban land, and high–low indicates isolated urban land. In this study, based on a 1 km × 1 km grid covering the entire FDB, we computed the area of urban land per grid cell for each study year. Then, the local Moran’s I index was estimated as follows, and we obtained the spatio-temporal distribution of the local Moran’s I per study year.

(4) $$I_{i}=\frac{v_{i}-\bar{v}}{\sigma^{2}}\sum_{j=1}^{n}\left[w_{ij}\left(v_{j}-\bar{v}\right)\right]\;\left(i\neq j\right),$$

where $v_{i}$ is the area of urban land in the grid cell *i*, $\bar{v}$ is the mean value of the areas of urban land with the sample number of *n*, $v_{j}$ is the area of urban land in other grid cells, $\sigma^{2}$ is the variance of the variable *v*, and $w_{ij}$ is the spatial weight that could be defined as the inverse of the distance among grid cells i and j or decided by the use of a defined distance band.

### 2.6. Urban Expansion Types

To investigate the spatio-temporal dynamics of the urban expansion types, we computed the LEI within the entire study area (Figure 1) for five time periods (i.e., 1960–1975, 1975–1986, 1986–1997, 1997–2009, and 2009–2015), which is defined as Formula (5). Such an index quantifies the spatial relationship between newly expanded patches and pre-growth urban patches [48]. Urban expansion type is defined as infilling for LEI > 50, edge-expansion for 0 < LEI ≤ 50, and spontaneous growth for LEI = 0. Then, the relative dominance of each expansion type over a given time period was estimated by the use of the Area Weighted Mean Expansion Index (AWMEI) as Formula (6) [48]. Higher values of AWMEI correspond to a more compact expansion of urban land, whereas smaller values correspond to more dispersal expansion of urban land.

(5) $$LEI=100\times \frac{A_{o}}{A_{o}-A_{v}}$$

where LEI represent the value of the land expansion index for a newly expanded urban patch, $A_{o}$ represents the intersection between the buffer zone with a specified distance around a newly expanded patch and old urban patch, and $A_{v}$ represents the intersection between the buffer zone and vacant land.

(6) $$AWMEI=\overset{N}{\underset{i=1}{\sum }}LEI_{i}\times \left(\frac{a_{i}}{A}\right)$$

where $LEI_{i}$ represents the value of landscape expansion index for the new urban patch i, $a_{i}$ is the area of the new urban patch i, and A represents the total area of the new urban patches.

### 2.7. Urban Expansion Efficiency

To measure how efficiently the urban land expanded, we computed the land consumption rate (*LCR*), population growth rate (*PGR*), and the ratio of *LCR* and *PGR* for five time periods (1960–1975, 1975–1986, 1986–1997, 1997–2009, and 2009–2015) as follows [32]:

(7) $$LCR=\frac{\ln\left(\frac{U_{t2}}{U_{t1}}\right)}{T},$$

(8) $$PGR=\frac{\ln\left(\frac{Popt2}{Popt1}\right)}{T}$$

(9) $$LCRPGR=\frac{LCR}{PCR},$$

where T, $U_{t1}$, $U_{t2}$, Popt1, and Popt2 represent the time period, the urban land area at the beginning time of the time period, the urban land area at the end time of the time period, the population at the beginning time of the time period, and the population at the end time of the time period, respectively.

## 3. Results

### 3.1. Urban Expansion Magnitude

**Figure 2:**
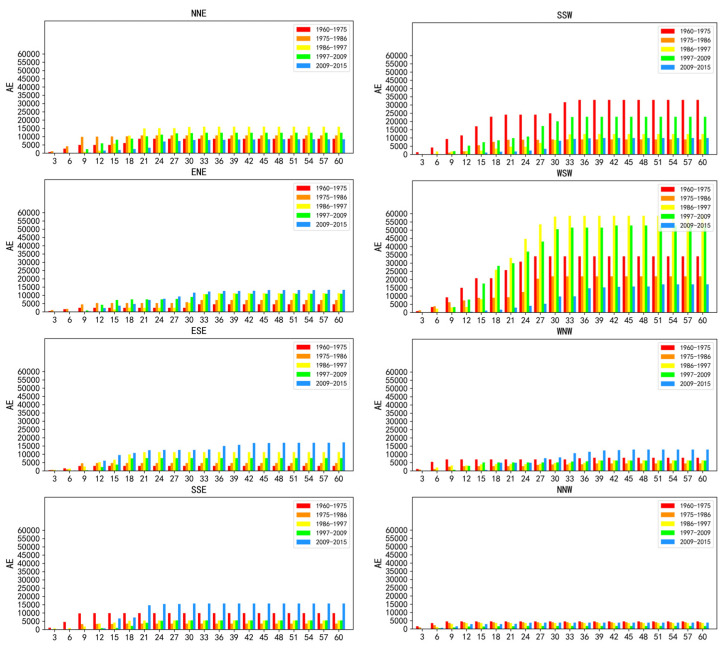
Annual expansion per direction across concentric rings during 1960–2015.

Table 3 lists the AE of the urban land in the entire study area for the five time periods. The highest and lowest value of AE were found in 1986–1997 and 1975–1986, respectively. Moreover, the AE per geographic direction across concentric rings for the five time periods are presented in Figure 2. Evidently, AE varied significantly among different directions and depended on the distance from the city center. The faster urban expansion could be found in the SSW and the WSW directions, in particular for the periods 1960–1975, 1986–1997, and 1997–2009.

### 3.2. Temporal Changes in Urban Land Patterns

**Figure 3:**
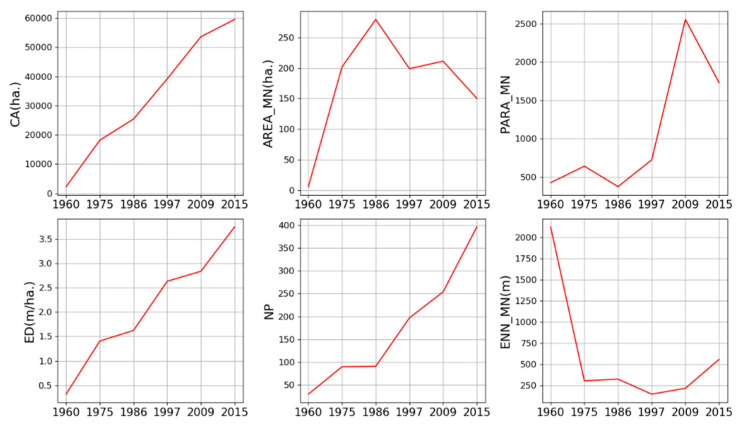
Temporal changes in landscape metrics in the FDB during 1960–2015.

Figure 3 presents the trends in the changes of urban land patterns in the entire study area during 1960–2015. CA, ED, and NP showed a rising trend that indicates urban land became more fragment and complex. However, ENN_MN sharply declined from 1960 to 1997, and it had a slight increase after 1997. This indicates that before 1997, urban land mainly expanded in the areas between the urban patches of 1960. After 1997, urban land expanded surrounding the pre-existing urban land patches of 1997, particularly in 2009–2015. AREA_MN reflects the changing trend in both CA and NP that sharply increased from 1960 to 1986, and then it had a slight decline.

**Figure A1:**
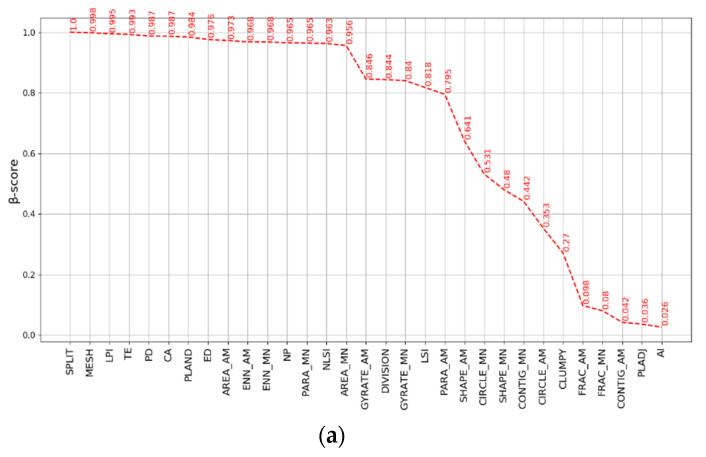

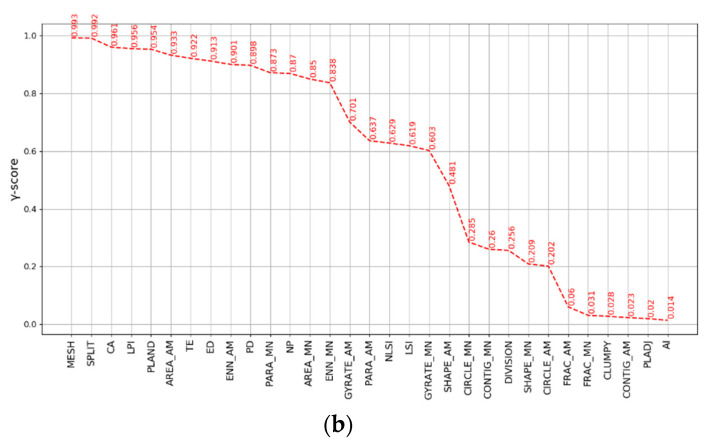
Landscape metrics computed based on the sectors of the concentric buffers–geographic directions system. (**a**) Mean β-score and (**b**) Mean γ-score reflect the capability of the landscape metrics to recognize the urban expansion patterns in the Federal District of Brazil over space and time, respectively.

Moreover, it is very important to verify whether landscape metrics adapt well to the multi-date urban expansion data and spatial sectors in the analysis of urban land patterns. Among the thirty-one metrics, less than half of the metrics had relatively high values (greater than 0.80) of both mean β-score and mean γ-score (Figure A1), including CA, AREA_MN, PARA_MN, ED, NP, and ENN_MN (Table 4). It indicates that they performed relatively consistently over space and time; that is, they adapted well to our multi-date urban expansion data and sectors used in this study. Such result ensures the interpretation of urban land patterns at different distances and in different directions using the six metrics. Nonetheless, only CA and NP were displayed hereafter as they are more intuitive to reflect the temporal changes in urban land at different distances and in different directions.

**Figure 4:**
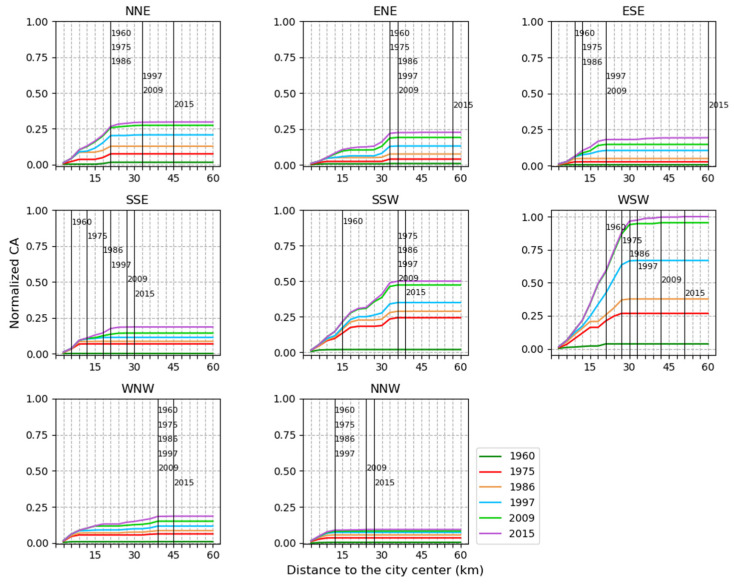
The total area of urban land in different directions and within concentric rings from 1960 to 2015. The x-axis represents the distances to the city center. The y-axis represents the normalized value of the metric. The black vertical line represents the distance where the metric value became stable for each year.

In terms of the area of urban land (Figure 4), a consistently increasing trend in CA values from 1960 to 2015 showed that urban expansion occurred in all eight directions, and the significant urban expansion could be observed in the SSW and WSW directions. Moreover, in each direction, urban expansion ended at a different distance from the city center during 1960–2015 (see the black vertical lines in Figure 4). For example, in the WSW direction, urban patches in 1960 were located within 21 km from the center, and they reached the distance of 52 km from the center in 2015.

**Figure 5:**
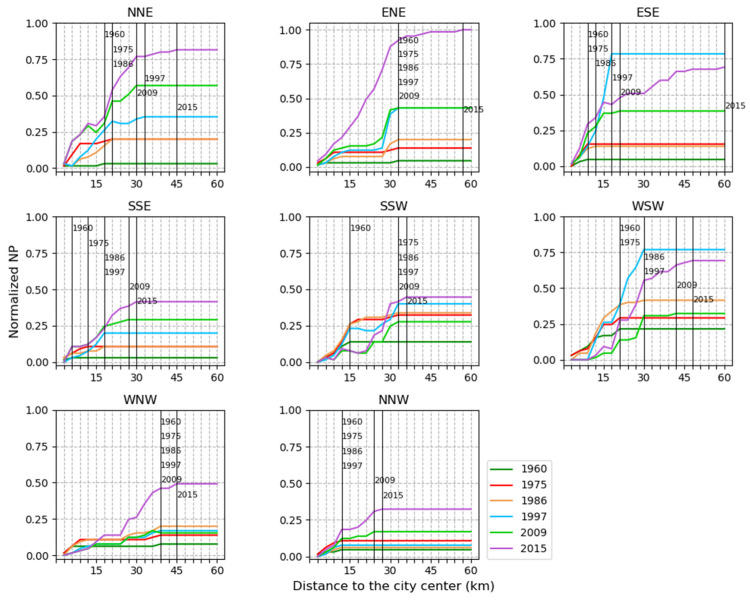
The number of urban patches in different directions and within concentric rings from 1960 to 2015. The x-axis represents the distances to the city center. The y-axis represents the normalized value of the metric. The black vertical line represents the distance where the metric value became stable for each year.

In terms of the subdivision of urban land (Figure 5), NP presented a consistently increasing trend from 1960 to 2015 for the NNE, ENE, and SSE directions. For the ESE, SSW, WSW, and WNW directions, NP increased from 1960 to 1997, decreased from 1997 to 2009, and then increased again from 2009 to 2015. For the NNW direction, there was a slight variation in NP values from 1960 to 1997, and NP presented an evident increase from 1997 to 2015.

### 3.3. Polycentric Urban Expansion

**Figure 6:**
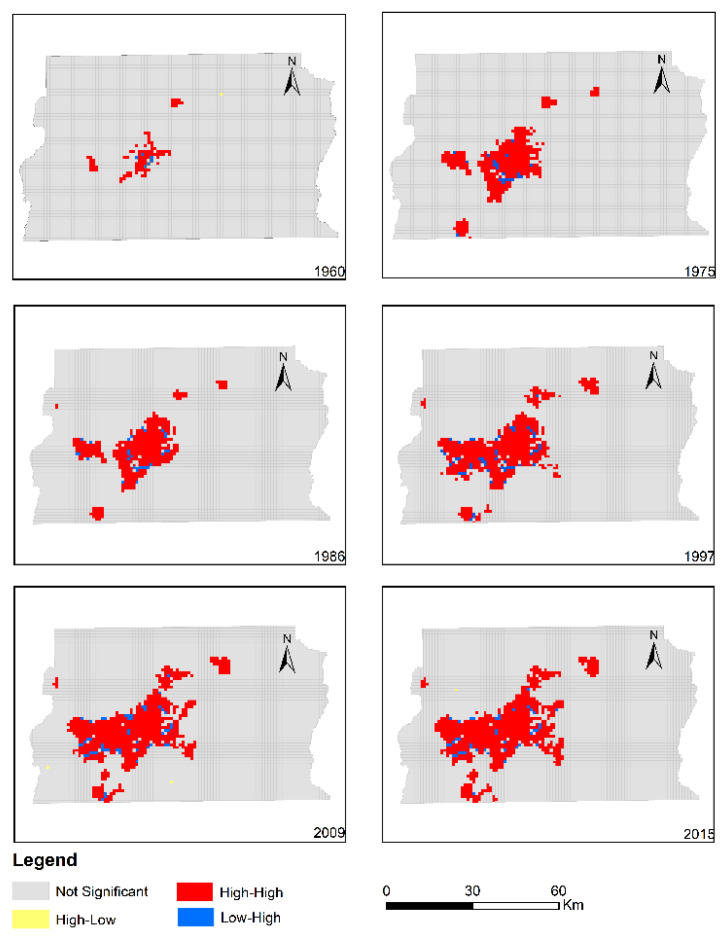
Spatio-temporal distribution of the local Moran’s I computed based on a 1 km × 1 km grid in the FDB.

Figure 6 presents the spatio-temporal distribution of the local Moran’s I computed based on a 1 km × 1 km grid covering the entire FDB. The results indicate that urban land in the FDB evolved in a polycentric way during 1960–2015. Moreover, urban expansion was characterized by the expansion of pre-existing urban land patches, and the emergence of new urban patches. The number of urban land clusters varied from three to five, with the main agglomeration of urban land in the middle of the FDB.

### 3.4. Spatio-Temporal Dynamics of Urban Expansion Types

**Figure 7:**
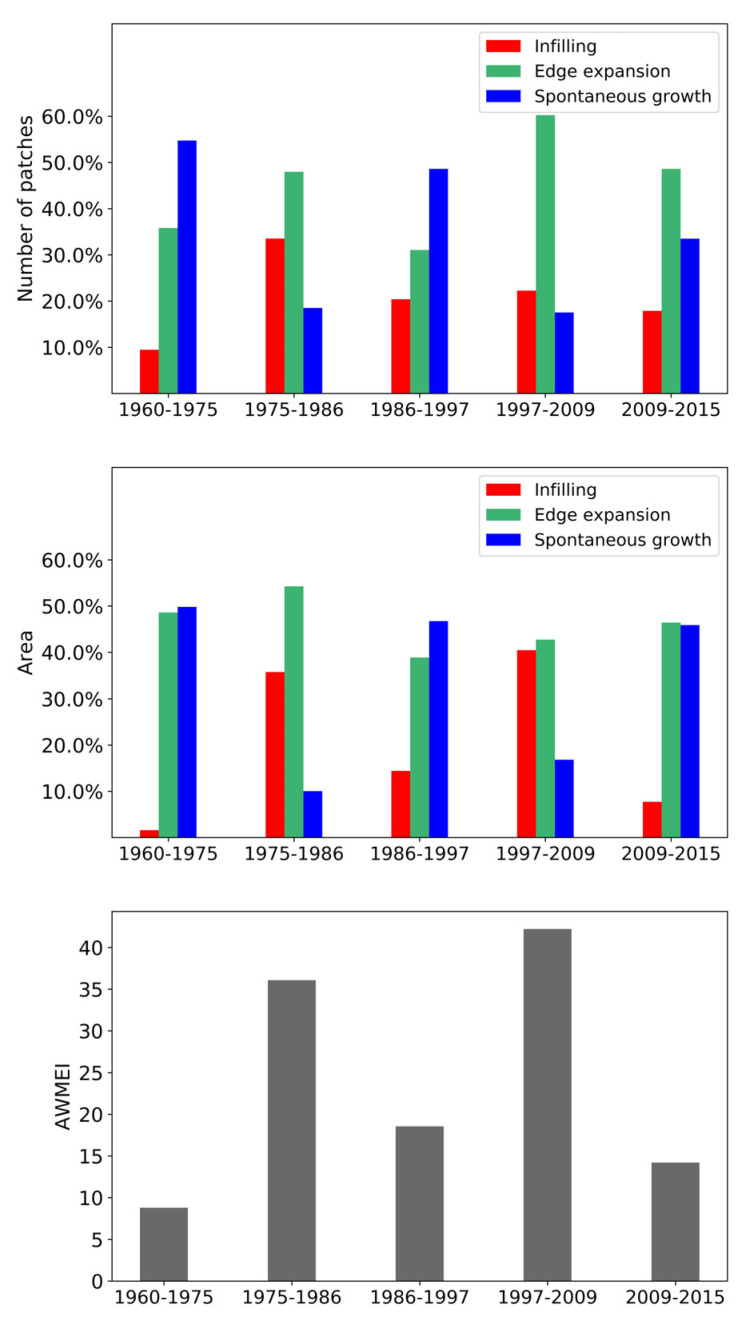
Temporal dynamics of urban expansion types and AWMEI values in the FDB from 1960 to 2015.

Figure 7 shows the quantitation of the three urban expansion types (i.e., infilling expansion, edge expansion, and spontaneous growth) in terms of the number and area of newly expended patches and the AWMEI value in the FDB during 1960–2015. The number and the area of new urban patches correspond to the frequency and extensiveness of urbanization, respectively, and they provide the complementary information on temporal dynamics of urban expansion types [49]. As presented in our study, the temporal dynamics of the three expansion types derived from the two analyses were consistent for each time period. Overall, edge expansion was the important contributor to urban expansion for all time periods, and the importance of infilling and spontaneous growth alternated. The dispersal and compact form of urban land appeared alternately according to the temporal changes in AWMEI values. Specifically, in 1960–1975, spontaneous growth and edge expansion dominated and the value of AWMEI is lowest during that time span. Compared with edge expansion, the number of newly expanded patches in the form of spontaneous growth was more than the ones in the form of edge expansion; it presented a similar status of the composition of urban expansion types in 1986–1997. The value of AWMEI was relatively small in this period, corresponding to a more dispersed urban land. In 1975–1986 and 1997–2009, edge expansion and infilling expansion were dominant, and the values of AWMEI were relatively high, representing a more compact urban land. In 2009–2015, the status of the urban expansion types was different from other time periods. Infilling expansion contributed the least, which was much less than the other types. A lower value of AWMEI indicated that urban land became more dispersed after 2009.

**Figure 8:**
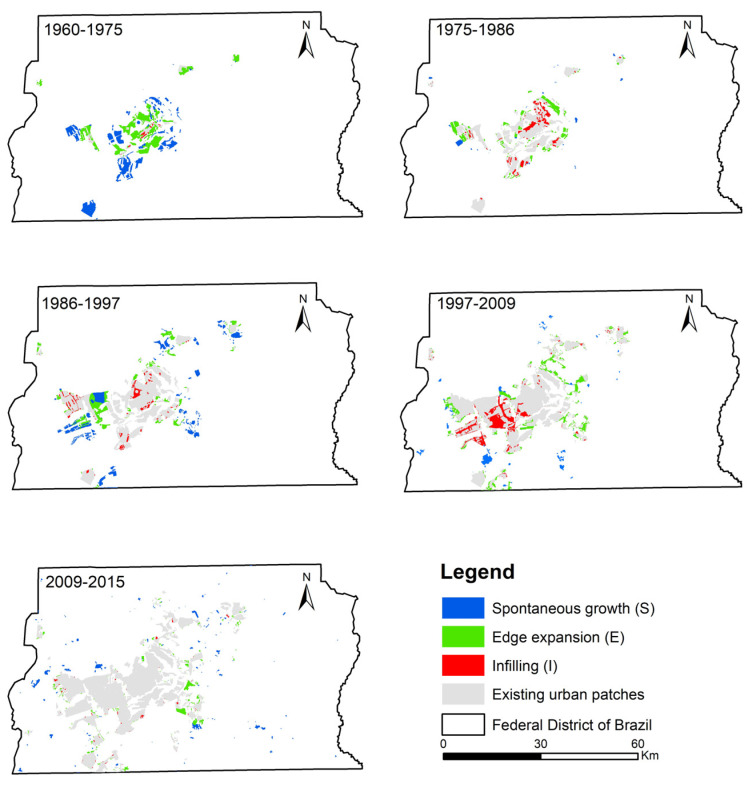
Spatial distribution of urban expansion types in the FDB during 1960–2015.

Figure 8 shows the spatial distribution of newly expended patches in each time period. Particularly, in 1960–1975, newly expanded patches scattered around the urban patches of 1960. In 1975–1986 and 1997–2009, newly expanded patches mostly linked the neighborhood patches or extended outward from old patches. In 2009–2015, newly expanded patches dispersed in this region.

### 3.5. Ratio of Land Consumption Rate to Population Growth Rate

Table 5 presents the LCR, PGR and the ratio of LCR to PGR for 1960–1975, 1975–1986, 1986–1997, 1997–2009, and the entire study period. Based on the results of LCRPGR, urban land expanded relatively faster than population growth in 1997–2009 and 2009–2015, and it enables catch up with the population growth over the entire study period (i.e., 1960–2015).

## 4. Discussion

This study explored the characteristics of urban expansion from scratch to urban agglomeration in the FDB that has taken place during 1960–2015. The results support the discussion of the suitability of diffusion–coalescence urban expansion theory and the impact of population growth and urban development policies on urban expansion in the FDB.

### 4.1. Suitability of the Diffusion—Coalescence Urban Expansion Theory in the FDB

Taking together, the temporal dynamics of CA, NP, AREA_MN, ENN_MN (Figure 3), and spatio-temporal shifts of each expansion type during 1960–2015 (Figure 7 and Figure 8), we identified the cyclical process of diffusion and coalescence in the FDB, with the diffusion phases in 1960–1975, 1986–1997 and 2009–2015, and the coalescence phases in 1975–1986 and 1997–2009 (Table 6). In 1960–1975, the increase in NP and AREA_MN and the decrease in ENN_MN were not consistent with their temporal dynamics proposed in urban expansion theory. However, urban expansion in the FDB started from multiple urban cores of 1960, and there were also the relatively large distances between these cores (Figure 8). Newly expanded patches scattered around these cores, and the area of newly expanded land, were much larger than the area of urban land in 1960 (Figure 8). These facts explain why AREA_MN increased and ENN_MN decreased with the increase in NP. Thus, a diffusion phase could be identified for this time period. In 1975–1986, NP was relatively stable and AREA_MN increased greatly. The new urban patches linked the neighborhood urban patches or extended outward from previous patches (Figure 8). These facts clearly indicated a coalescence phase of urban expansion. In 1986–1997, the increase in NP, the decrease in AREA_MN, the spatial distribution of new urban patches (Figure 8), and their contribution in the form of edge expansion and spontaneous growth clearly pointed out a diffusion phase of urban expansion in this period. In 1997–2009, NP increased and AREA_MN increased slightly. Moreover, edge-expansion and infilling expansion were more pronounced in this period. The two results are opposed. According to the value of AWMEI, a high value in this period indicated that new urban patches are closer to existing urban patches. Therefore, we preferred a coalescence phase in this period. In 2009–2015, the changing trend in NP and AREA_MN is similar to the period 1986–1997. Moreover, ENN_MN increased, and new urban patches of a small size that dispersed in the FDB (Figure 8) mainly contributed in the form of edge expansion and spontaneous growth. These facts indicated a typical diffusion phase of urban expansion in this time period.

### 4.2. Some Implications for Sustainable Urban Expansion in the FDB

Regarding to driving factors of urban expansion, this study solely considered the population growth and urban development policies due to data availability in such a long time period (i.e., 1960–2015). The population had a very significant (*p*-value < 0.001) correlation with the area of urban land, with a Pearson coefficient of 0.995. In fact, compared with other regions in Brazil, the high income per capita, quality of life, and access to public services in the FDB attracted the migrants in the past [50], and the construction of a series of peripheral urban nuclei in this region solved the housing problem [37]. Together with the results of SDG indicator 11.3.1 (Section 3.5), it was found that population growth is an important driver of urban expansion in the FDB, and the urban expansion rate is greater than the population growth rate during 1960–2015. However, the land-use efficiency in the FDB is smaller than the average value of cities worldwide, with the LCRPGR equal to 1.5.

**Figure 9:**
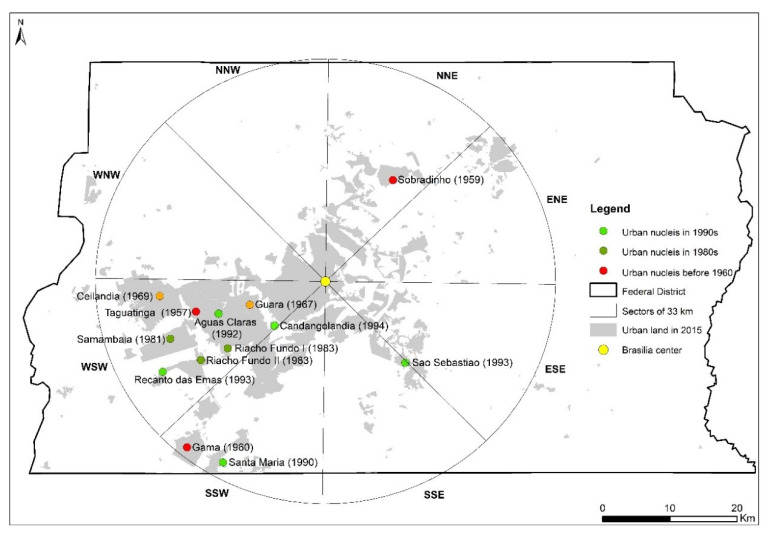
Location of the peripheral urban nucleus around Brasilia created from 1960 to 2015. Source: State Secretariat for Housing and Urban Development of the Federal District (SEDUH-DF).

The urban structure patterns could be explained by the time and place of urban development policies. An important study summarized the main urban development policies implemented in the FDB, and described their association with the evolution of the spatial structure of urban land over time [37]. Some important points were highlighted (Figure 9): (1) Brasilia was created as the new national capital in 1960. Three urban nuclei (i.e., Taguatinga, Sobradinho, and Gama) were already established before 1960, and two other urban nuclei (i.e., Guara and Ceilandia) were developed in the 1960s. These urban nuclei were created to accommodate workers and inhabitants from illegal settlements. (2) The sectors in the WSW and SSW directions were regarded as the main zone of urban expansion as the nuclei, such as Guara, Ceilandia, Samambaia, Riacho Fundo I, Riacho Fundo II, Santa Maria, Agua Claras, and Recanto das Emas were created around Brasilia in the 1980s and 1990s to address the housing shortage. (3) Land-use plans, such as PDOT-1992, PDOT-1997, and PDOT-2009, reinforced the development of urban nuclei, by regarding Taguatinga as a complementary center of Brasilia, and creating the industrial poles in Santa Maria, Guara, and Recanto das Emas.

Our findings permit to provide some implications for urban expansion and local sustainable development in the FDB. For example, recent urban expansion is in the stage of diffusion, and it should promote the policies to encourage the compact urban growth within sustainable development thinking. Furthermore, historical population growth and urban development policies form the basis of urban sustainability, and urban expansion faster than population growth often leads to a low urban population density. This implies the importance of strategies of population control and improving the efficiency of urban land use. Future studies could focus on the urban expansion in recent years and consider more aspects of urban sustainable development in terms of urban form and urban expansion.

## 5. Conclusions

This study first provides a retrospect of urban expansion in the FDB from scratch to urban agglomeration in the past 60 years, in terms of urban expansion magnitude, urban land patterns, urban development modes, urban expansion types, and land-use efficiency. It could be found that urban land continuously expanded in a polycentric way, and it became more and more fragmented and complex. Edge expansion was the important contributor, and the importance of infilling and spontaneous growth alternated. Urban land expanded faster than population growth.

Then, urban expansion theory adapted well the urban expansion from scratch to urban agglomeration in the FDB, with an alternation in the diffusion and coalescence phases. Population growth was positively related to urban expansion, and urban development policies related to the creation and development of a series of peripheral urban nuclei affected urban expansion in this region. This study provides not only new evidence of urban expansion but also some implications within sustainable urban development thinking, including encouraging compact urban expansion, controlling population growth, and improving the efficiency of urban land use.

## Figures and Tables

**Table 1:** Spatial scales and urban expansion analysis used in this study. ✓ represents the analysis was implemented, and X represents the analysis was not implemented.

Urban Expansion Analysis	Spatial Scales		Description
	Sectors	FDB	
Urban expansion magnitude	✓	✓	Quantifying the area of annual expansion of urbanized land
Urban land patterns	✓	✓	Quantifying the compositional and configurational patterns of urban land
Urban expansion modes	X	✓	Identifying the expansion modes: monocentric or polycentric
Urban expansion types	X	✓	Identifying the expansion types of newly urban land: Infilling expansion, edge expansion and spontaneous growth
Urban expansion efficiency	X	✓	Indicating whether the urban expansion in the FDB is inclusive and sustainable

**Table 2:** Landscape metrics used in this study.

Metric (Abbreviation)	Description Adapted from [41]	Units	Range
Class area (CA)	The sum of the area of urban land patches in the analysis unit	Hectares	≥0
Mean patch area (AREA_MN)	The average area of urban land patches in the analysis unit	Hectares	≥0
Mean perimeter-area ratio (PARA_MN)	Simple ratio of perimeter to area for each urban land patch in the analysis unit	None	≥0
Edge density (ED)	Total length of urban land edges in the computation unit, per hectare	Meters/ hectare	≥0, no limit
Number of patches (NP)	Total number of urban land patches in the analysis unit	None	>0
Mean Euclidean neighbor distance (ENN_MN)	Average distance between two urban land patches in the computation unit, based on the nearest cell center-to-center	Meters	≥0, no limit

**Table 3:** Annual expansion in the FDB during 1960–2015.

Time Periods	Annual Expansion (km^2^)
1960–1975	10.6
1975–1986	6.5
1986–1997	12.5
1997–2009	12.1
2009–2015	10.3

**Table 4:** Mean β-score and mean γ-score of the landscape metrics used in this study.

Landscape Metrics	Mean β-Score	Mean γ-Score
CA	0.987	0.961
AREA_MN	0.956	0.850
PARA_MN	0.965	0.873
ED	0.976	0.913
NP	0.965	0.870
ENN_MN	0.968	0.838

**Table 5:** Land consumption rate, population growth rate, and land-use efficiency in the FDB during 1960–2015.

Time Periods	LCR	PGR	LCRPGR
1960–1975	0.120	0.138	0.867
1975–1986	0.061	0.025	2.510
1986–1997	0.041	0.045	0.925
1997–2009	0.029	0.026	1.126
2009–2015	0.023	0.017	1.337
1960–2015	0.062	0.059	1.047

**Table 6:** Summary of the temporal dynamics of landscape metrics, urban growth types, and their abundance and urban expansion phases. ↑, ↓, and — indicate increase, decrease, and relatively stable, respectively. E, S, and I represent edge-expansion, spontaneous growth, and infilling, respectively.

Time Periods	CA	NP	AREA_MN	ENN_MN	Urban Expansion Types	AWMEI	Urban Expansion Phases
1960–1975	↑	↑	↑	↓	E + S	Low	Diffusion
1975–1986	↑	-	↑	—	E + I	High	Coalescence
1986–1997	↑	↑	↓	↓	E + S	Low	Diffusion
1997–2009	↑	↑	—	↑	E + I	High	Coalescence
2009–2015	↑	↑	↓	↑	E + S	Low	Diffusion

## Appendix A

Among the thirty-one landscape metrics evaluated using β-score and γ-score, and only fourteen metrics had relatively high values (i.e., greater than 0.80) of both mean β-score and mean γ-score (Figure A1). Thus, we encourage the evaluation of landscape metrics in further studies of urban expansion, especially using different spatial scales and multi-date urban expansion data.
